# Isolation and molecular characterization of fowl aviadenovirus associated with inclusion body hepatitis from poultry in Banten and West Java, Indonesia

**DOI:** 10.14202/vetworld.2020.1940-1946

**Published:** 2020-09-22

**Authors:** Otto Sahat Martua Silaen, Sri Murtini, Joko Pamungkas, Christian Marco Hadi Nugroho

**Affiliations:** 1 Department of Animal Infectious Disease and Veterinary Public Health, Faculty of Veterinary Medicine, IPB University, Bogor, Indonesia; 2 Animal Health Diagnostic Laboratory, PT Medika Satwa Laboratories, Bogor, Indonesia

**Keywords:** fowl aviadenovirus, hexon gene, Indonesia, molecular characterization, poultry

## Abstract

**Background and Aim:**

Fowl avidenoviruses (FAdVs) are generally considered ubiquitous, but certain serotypes and strains are known to be associated with primary diseases, such as inclusion body hepatitis (IBH). Since 2018, the outbreak of IBH has been reported in part provinces of Indonesia. This study aimed to isolate and molecularly characterize the FAdV from Banten and West Java Provinces of Indonesia and described the phylogenetic relationship with the FAdV that has been characterized in other countries.

**Materials and Methods:**

A total of 25 FAdV archive samples have been collected from January to August 2019 from clinical cases of FAdV infection in Banten and West Java Provinces, Indonesia. Collected samples were inoculated in 10-day-old specific-pathogenic-free chicken embryonated eggs. Hexon gene of FAdV was detected using polymerase chain reaction (PCR) with a primer set from previous study. To gain a better understanding of the FAdV genetic properties and construct the phylogeny tree, the PCR products were sequenced and subjected to a BLAST search and inferred using the neighbor-joining method by bootstrap test 1000×.

**Results:**

FAdV-D and FAdV-E are present in Banten, Indonesia. The phylogenetic analysis of 850 nucleotides that encode 289 amino acid of the partial hexon gene shows that the isolates Broiler/MSL/Ciputat-149/18, Broiler/MSL/Lebak-151/18, and Broiler/MSL/Ciputat-29/19 have 100% homology with FAdV-E TR/BVKE/R/D-1 from Turkey, whereas the isolates Layer/MSL/Ciputat-20/19 and Broiler/MSL/Ciputat-30/19 have 100% homology with FAdV-D strain 685 from Canada.

**Conclusion:**

The present study provides updates of the circulating FAdV in commercial poultry flocks in Banten and West Java Provinces, Indonesia. Since the FAdV vaccine was unavailable in Indonesia, this result might be used as guidance to select a proper FAdV vaccine strain. Our result indicates that at least two FAdV species were circulating among poultry in Banten and West Java Provinces, Indonesia; they are FAdV-D and FAdV-E.

## Introduction

Adenoviruses are members of the family Adenoviridae, non-enveloped with icosahedral nucleocapsids that contain a double-stranded DNA. They are divided into the following five genera: Atadenovirus, Aviadenovirus, Ichtadenovirus, Mastadenovirus, and Siadenovirus [1]. Many poultry species such as chickens, geese, ducks, turkeys, and pheasants can be infected by Aviadenovirus. Various diseases caused by aviadenovirus include egg drop syndrome, hemorrhagic enteritis, pheasant marble spleen disease, quail bronchitis, and inclusion body hepatitis (IBH) [2].

IBH is one of the diseases caused by the fowl avidenoviruses (FAdVs) infection, which acutely attacks young chickens between 1 and 5 weeks. The disease is characterized by an increase in chicken mortality which reaches its peak on the 4^th^ or 5^th^ day after infection. The main characteristic of this disease appears in an enlarged liver, bleeding, and necrosis. The presence of intranuclear basophilic inclusion bodies in hepatocyte cells was the main microscopic lesions [3]. The death rate caused by IBH varies usually below 10%, but can be more than 30% [4]. Since the first case identified in the United States in 1963, IBH disease has become a serious threat to the poultry industry worldwide. In some countries such as Pakistan, India, Korea, Canada, the United States, Hungary, Japan, and China, IBH disease has caused considerable economic losses [5].

The transmission of FAdV can occur horizontally or vertically. Horizontal transmission occurs through feces, food, water, and virus-contaminated environments, whereas vertical transmission occurs through eggs from breeder chicken infected with FAdV-4 and FAdV-8 [6]. Infected chickens that shown no clinical symptoms are thought to be the source of the spread of IBH disease, especially to chickens with immune system disorders. The main species that cause IBH infection in chickens are FAdV-D and FAdV-E with serotypes 7, 8a, 8b, and 11 [7]. Over the past few years, there have been complaints of the presence of IBH or hepatitis hydropericardium syndrome in Indonesia. The outbreak of the syndrome started in 2018 [8].

This study aims to isolate and determine FAdV strain and genetic characterization of hexon gene of FAdV isolated from poultry in Banten and West Java Provinces, Indonesia. The results obtained are expected to provide updated information about the presence of the FAdV in this region, as well as providing molecular character data and genetic relationships with the FAdV in other countries so that it can be a reference for the selection of seed virus IBH vaccine.

## Materials and Methods

### Ethical approval

No live animals were used in the present study. Therefore, no ethical approval was necessary. This study was performed based on the regulations for Research in Animal Health of Indonesian Law on Livestock and Animal Health (UU/18/2009, article 80).

### Study period and location

This research was conducted from March 2019 to January 2020 in the Virology Laboratory Research and Development Unit (R&D) of PT Medika Satwa Laboratories, Bogor and in the Integrated Laboratory Department of Animal Infectious Disease and Veterinary Public Health, Faculty of Veterinary Medicine, IPB University, Bogor, Indonesia

### Samples

A total of 25 samples were an archival collection of PT Medika Satwa Laboratories, West Java, Indonesia, that isolated from problematic flocks showing IBH such as clinical symptoms and decrease in production. The samples were collected from commercial poultry flocks in some districts of Banten and West Java Provinces: Bogor (n=9), Ciamis (n=2), Lebak (n=3), Sukabumi (n=3), Subang (n=2), and Tanggerang (n=6). The owner of each sample was given an informed consent and agreed that the sample was used in this study.

### Virus propagation

Virus propagation was done by inoculated 0.2 ml filtered suspension (20% W/V) of pooled tissue samples from one farm to the allantoic cavity of three 10-day-old fertilized specific-pathogen-free (SPF) chicken eggs. The inoculum contains 200 μg/ml penicillin and 100 μg/ml streptomycin. The SPF chicken eggs that have been inoculated were incubated at 37°C and observed for a maximum 6 days [4]. The negative embryo control was three 10-day-old fertilized SPF chicken eggs inoculated by 0.2 mL sterile phosphate-buffered saline. The embryo was observed for any signs of abnormalities, such as bleeding and death. The allantoic fluid was harvested from the SPF chicken eggs that may be dead and live. Furthermore, allantoic fluid was pooled and processed for DNA extraction and polymerase chain reaction (PCR).

### DNA extraction and PCR

The entire DNA allantoic fluid was extracted using Viral Nucleic Acid Extraction Kit II (Geneaid, Taiwan) according to the manufacturer’s protocol. The DNAs were dissolved in 50 μL nuclease-free water and directly used for subsequent PCR or stored at –20°C. The FAdV hexon gene fragment was amplified using a published hexon A and hexon B primer set list in Table-1 [9] with expected PCR product size that is 897 bp. PCR amplification reaction was carried out in 50 μL containing 5 μL DNA template, 25 μL KAPA2G Fast ReadyMix (Sigma-Aldrich, USA), 5 μL primer (500 nM), and 15 μL DEPC-H2O. An amplification reaction was carried out with thermal profile at a pre-denaturation condition of 95°C for 30 s, followed by 25 cycles of annealing 56°C for 30 s, extension 72°C for 60 sec, and concluded with an additional final extension 72°C for 5 min. Amplified samples were analyzed by electrophoresis in 1.5% agarose gel and stained by ethidium bromide. A molecular weight marker with 100 bp (Geneaid, Taiwan) was used as a standard size [10,11].

**Table-1:** Hexon gene fragments and primer sequences used for PCR identification.

Gene	Primer sequence	Size (bp)	Annealing temperature	References
Hexon	(F) 5′- CAARTTCAGRCAGACGGT -3′	897	56°C	[9]
	(R) 5′- TAGTGATGMCGSGACATCAT -3′			

### Sequencing and analysis

About 40 μL each of PCR product of amplified hexon gene were sent for sequencing (First Base, Malaysia) and sequenced from both directions using BigDye® Terminator version 3.1 Cycle Sequencing Kit (Thermo Fisher Scientific, USA) with forward hexon A and reverse hexon B primer. Sequence alignment was conducted using MEGA 7 [12]. All standard FAdV sequences were downloaded from GenBank and compared with the sample nucleotide sequence. The phylogeny tree was inferred using the neighbor-joining method and tested using the bootstrap test of 1000 replicates.

## Results

**Figure-1:**
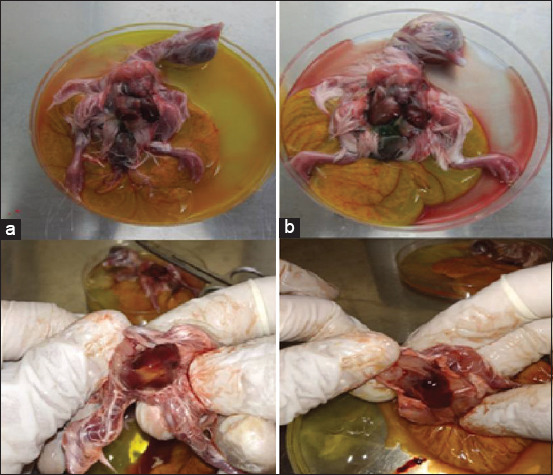
The pathologic lesions of the embryo, code a is the embryo inoculated by samples suspension, while code b is a control embryo inoculated by sterile PBS.

The propagation results showed that 9 of the 25 samples in the present study caused pathological lesions in the kidney, liver, heart, and skeletal muscles of the chicken embryo, but no dead chicken embryo was found until 144 h post-inoculation. The observation of the chicken embryo showed imperfect growth compared to the control embryo. Most pathologic lesions that were found are swelling/hemorrhage of the liver with a brownish-yellow color with foci necrotic and a soft consistency of the heart with hydropericardium. Enlarged or pale kidneys were also observed in some inoculated embryos. No lesions were found in the control embryo (Figure-1). The summary of origin, age of the affected chickens, pathologic lesions, and PCR result are presented in Table-2.

**Table-2:** List of origin, age of affected chickens, embryo pathologic lesions, and PCR result.

No.	Samples code	Province	Chicken age (days)	Embryo mortality	Embryo pathologic lesion	PCR result
1	Broiler/MSL/Ciputat-21/19	Banten	21	Live	Normal	Negative
2	Broiler/MSL/Ciputat-29/19	Banten	30	Live	Yellowish and hemorrhagic liver	Positive
3	Broiler/MSL/Ciputat-30/19	Banten	35	Live	Hepatomegaly and hemorrhage	Positive
4	Broiler/MSL/Ciputat-166/19	Banten	30	Live	Normal	Negative
5	Broiler/MSL/Ciputat-149/18	Banten	25	Live	Kidney swelling, hemorrhagic liver	Positive
6	Broiler/MSL/Lebak-151/18	Banten	17	Live	Petechiae in skeletal muscle	Positive
7	Broiler/MSL/Lebak-322/19	Banten	26	Live	Normal	Negative
8	Broiler/MSL/Sukabumi-165/19	West Java	39	Live	Normal	Negative
9	Broiler/MSL/Ciamis-240/19	West Java	25	Live	Normal	Negative
10	Broiler/MSL/Bogor-127-19	West Java	29	Live	Pale hepatomegaly	Negative
11	Broiler/MSL/Bogor-318/19	West Java	26	Live	Yellowish liver	Negative
12	Broiler/MSL/Bogor-378/19	West Java	26	Live	Normal	Negative
13	Broiler/MSL/Subang-324-19	West Java	17	Live	Normal	Negative
14	Broiler/Bogor-IP1/19	West Java	31	Live	Normal	Negative
15	Broiler/Bogor-IP2/19	West Java	31	Live	Normal	Negative
16	Broiler/Bogor-IP3/19	West Java	31	Live	Normal	Negative
17	Broiler/Bogor-IP4/19	West Java	31	Live	The liver is brittle and looks yellow	Negative
18	Broiler/Bogor-IP5/19	West Java	31	Live	Normal	Negative
19	Layer/MSL/Ciputat-20/19	Banten	175	Live	Yellowish liver	Positive
20	Layer/MSL/Lebak-450/19	Banten	210	Live	Normal	Negative
21	Layer/MSL/Sukabumi-83/19	West Java	217	Live	Kidney swelling, hemorrhage in the liver	Positive
22	Layer/MSL/Sukabumi-164/19	West Java	203	Live	Normal	Negative
23	Breeder/MSL/Ciamis-255/19	West Java	231	Live	Normal	Negative
24	Breeder/MSL/Ciputat-193/19	Banten	245	Live	Normal	Negative
25	Breeder/MSL/Subang-333-19	West Java	231	Live	Normal	Negative

**Figure-2:**
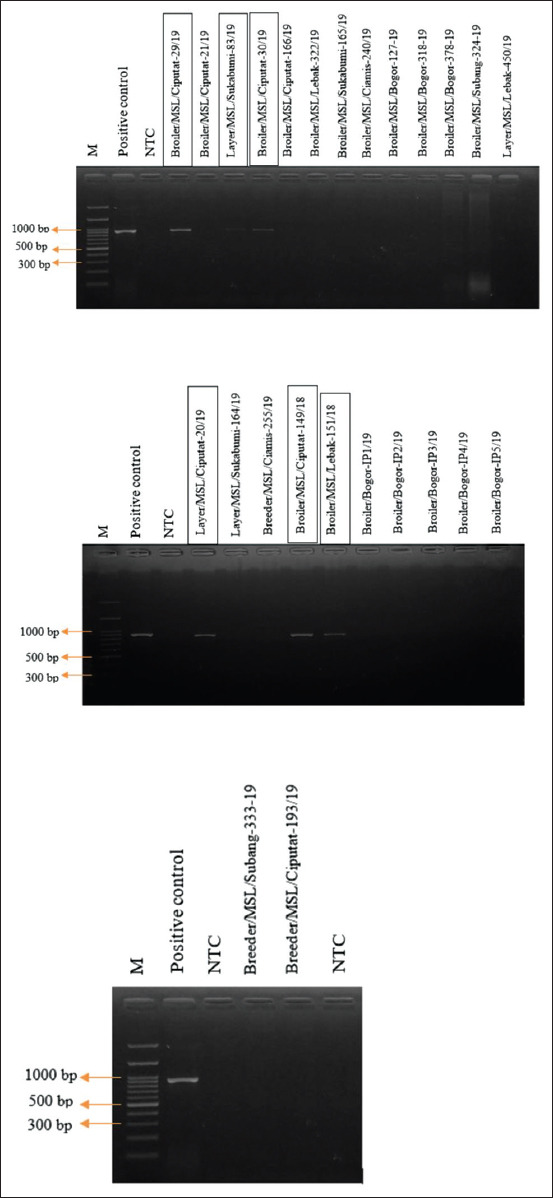
Amplification of hexon gene fragments determinants on agarose gel approximate 897 bp. M: 100 bp DNA ladder. NTC: Non-template control. The positive isolates from the samples we collected marked with rectangle shape in isolates name.

PCR test results from the whole DNA of allantoic fluid SPF chicken eggs inoculated with field samples of suspected FAdV infection revealed amplifications of a hexon gene fragment as expected (897 bp) on 6 of 25 samples (Figure-2). The positive control was DNA from the first specific FAdV case obtained, while the negative control was non-template control (NTC). Six samples that showed positive results were three isolates from broiler farms in Ciputat, one isolate from layer farms in Ciputat, one isolate from layer farms in Sukabumi, and one isolate from broiler farms in Lebak. Of the six positive results, five have been sequenced and available in GenBank with accession numbers of MT104454-MT104458.

**Figure-3:**
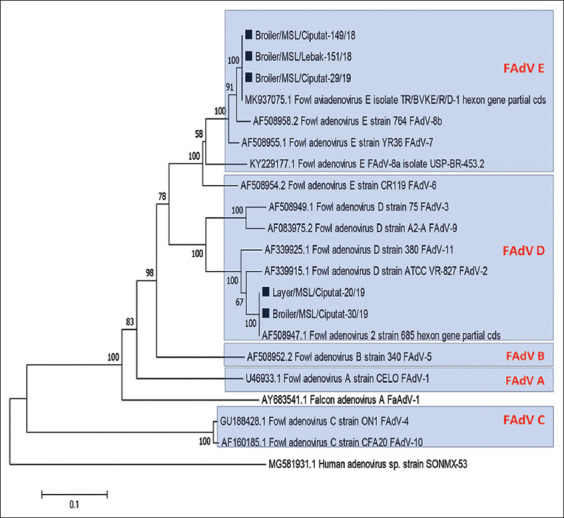
Phylogenetic tree of FAdV isolated from positive samples in Banten. Five isolates in the present study were marked with ■ while other isolates were available at GenBank with the accession number attached. The phylogenetic tree was constructed using the neighbor-joining method with 1000 bootstraps replicating on Mega 7 [22]. Sequence of a human adenovirus sp. SONMX-53 accession number MG581931 is coanalyzed as an out group.

Hexon gene fragments result from five FAdV isolates obtained 850 nucleotides which encode about 289 amino acids. The homological analysis was performed by pairwise comparison which compares the nucleotide sequences of the hexon gene fragments from isolates in the present study with other FAdVs available at GenBank. The results of the phylogenetic analysis of the hexon gene fragments from FAdVs in the present study and other FAdVs available at GenBank database are presented in Figure-3. The result shows that three isolates classified into the same group, namely, Broiler/MSL/Ciputat-149/18, Broiler/MSL/Lebak-151/18, and Broiler/MSL/Ciputat-29/19. The three isolates clustered to FAdV-E have 100% homology with TR/BVKE/R/D-1 (Accession No. MK937075) and 97% homology with Strain 764 which is FAdV-8b (Accession No. AF508958). The other two isolates Layer/MSL/Ciputat-20/19 and Broiler/MSL/Ciputat-30/19 classified into FAdV-D have 100% homology with strain 685 (Accession No. AF508947) and 95.7% homology with P7-A FAdV-2 (Accession No. AF339915). The summary of homological analysis is presented in Table-3.

**Table-3:** List of homological analysis.

Virus species	Isolate/strain	Homology				

		1	2	3	4	5
FAdV A	Falcon adenovirus A	59.7	59.7	59.2	59.7	59.2
FAdV A	CELO FAdV-1	67.4	67.4	64.2	67.4	64.2
FAdV B	Strain 340 FAdV-5	73.6	73.6	70.2	73.6	70.2
FAdV C	ON1 FAdV-4	38.5	38.5	37.1	38.5	37.1
FAdV C	CFA20 FAdV-10	38	38	36.9	38	36.9
FAdV D	ATCC VR-827 FAdV-2	75.4	75.4	95.7	75.4	95.7
FAdV D	Strain 75 FAdV-3	73.8	73.8	80.4	73.8	80.4
FAdV D	A2-A FAdV-9	73.8	73.8	82.2	73.8	82.2
FAdV D	Strain 380 FAdV-11	74.7	74.7	93.2	74.7	93.2
FAdV D	Strain 685	76.1	76.1	100	76.1	100
FAdV E	CR119 FAdV-6	88.4	88.4	75.6	88.4	75.6
FAdV E	YR36 FAdV-7	96.6	96.6	75.2	96.6	75.2
FAdV E	USP-BR-453.2 FAdV-8a	87.9	87.9	73.8	87.9	73.8
FAdV E	764 FAdV-8b	97	97	75.2	97	75.2
FAdV E	TR/BVKE/R/D-1	100	100	76.1	100	76.1
HAdV	SONMX-53	25.3	25.3	25.1	25.3	25.1
FAdV E	Broiler/MSL/Ciputat-149/18 ^(1)^	/	100	76.1	100	76.1
FAdV E	Broiler/MSL/Lebak-151/18 ^(2)^	100	/	76.1	100	76.1
FAdV D	Layer/MSL/Ciputat-20/19 ^(3)^	76.1	76.1	/	76.1	100
FAdV E	Broiler/MSL/Ciputat-29/19 ^(4)^	100	100	76.1	/	76.1
FAdV D	Broiler/MSL/Ciputat-30/19 ^(5)^	76.1	76.1	100	76.1	/

## Discussion

The present study confirmed the presence of FAdVs in Banten and West Java Provinces, Indonesia. We made confirmation after inoculation of the liver sample suspension from suspect field cases into fertilized 10-day-old SPF chicken eggs, PCR test, and sequencing of specific FAdV hexon gene fragments. The pathological characteristics of some embryos inoculated with the suspension of liver samples have the same lesions according to the statement of Dutta et al. [13], who said that a chicken liver suffering from IBH will look pale to yellowish, swollen, brittle, and have necrotic. Viral propagation in the present study was carried out to reproduce and isolate the virus from the chicken’s liver suspect with IBH and fulfill the second Koch’s postulate rule where the microorganism can be isolated into pure cultures [14].

FAdV infection has been reported in various countries, most cases of FAdV attack broiler chickens aged between 2 and 5 weeks [7,8,15-17]; however, FAdV infections in layers and breeders are not uncommon [3,18]. The results of this study did not distinguish observations and final confirmation between types of broiler, layer, or breeder farms.

We confirmed the presence of FAdV in 6 of 25 samples by PCR that showed positive results. Some samples, namely, Broiler/MSL/Bogor-127-19, Broiler/MSL/Bogor-318/19, and Broiler/Bogor-IP4/19, caused pathological lesions in the embryo, but showed negative results by the PCR test. The other infections that showed pathologic lesions in the liver and kidneys like FAdVs infection are due to the presence of aflatoxin in the sample being tested. Aflatoxin is the most common factor associated with IBH because the clinical sign and immunosuppression effect are similar to FAdV infection [19].

The primer set used in the present study can amplify DNA from the five of FAdVs species. Negative results from the samples are strongly suspected due to the absence of specific FAdV or insufficient amount of DNA template so that it is below the detection limit [15]. To increase the amount of DNA virus (titer), it should be able to do the reinoculation twice into new SPF eggs before being discarded as negative [20]. Detection results with PCR continued to sequencing. Of the six positive isolates, there was one isolate that could not be sequenced (Layer/MSL/Sukabumi-83/19). There are several reasons that sequencing cannot be done, one of them is inadequate concentration of the template, which can be seen through the band on the agarose gel. To increase the template concentration, it is generally possible to do a viral passage before molecular testing.

Data from the results of the present study have not been able to determine the source of spread nor the origin of FAdV infections in Indonesia. Wibowo *et al*. [8] reported that the highest number of FAdV cases in Indonesia between 2018 and 2019 came from East Kalimantan, East Java, and South Kalimantan, where the species that were infected are the same as FAdV found in the present study with homology 98.9-100%. From these results, we conclude that the FAdVs associated with IBH in several provinces in Indonesia are the same strain.

The investigation by Wibowo *et al*. shows that the spread of FAdV occurs without a clear epizootiological relation [8]. Similar reports regarding the source of the FAdV spread also described in Japan by Mase and Nakamura [21]. FAdVs are known to be transmitted horizontally between flocks through the fecal–oral route and vertically in breeder chickens [16], but in the present study, we did not find any breeder farms infected with FAdV. Breeder farms that supply a 1-day-old chicken to some broiler farms in Ciputat may be the source of infection, but this needs further investigation.

Some hypotheses related to the cause of entry FAdV include migration of wild birds. However, there was only one report related to wild birds as a reservoir in the spread of FAdV available in PubMed [22] to prove this hypothesis. Research on the existence of FAdV in wild birds in Indonesia is needed. Some outbreak reports of FAdV in various countries include: Iran reported as a result of infection with FAdV-C and FAdV-E [23]; Japan caused by FAdV-A and FAdV-D [21]; Canada by FAdV-D and FAdV-E [7]; and India by FAdV-B, -C, -D, and -E [15].

In the Southeast Asia region, FAdV-D has been reported in Thailand [24], while FAdV-E has been reported in Malaysia [25]. Considering the close geographical distance, FAdV-D and FAdV-E detected in Indonesia may come from neighboring countries. Further phylogeography studies need to be done to estimate the origin of the FAdV circulating in Indonesia.

## Conclusion

FAdV associated with IBH was successfully isolated from several chicken farms in Banten and West Java Provinces, Indonesia. Phylogenetic analysis revealed two FAdV species circulating in the Banten and West Java regions, which are FAdV-D and FAdV-E that belonged to serotypes 2 and 8b. The FAdV in this study was genetically identical to the previously isolated FAdV from Indonesia and the FAdV from Turkey and Canada.

## Authors’ Contributions

OSMS designed the study and drafted the manuscript under the supervision of SM and JP. CMHN collected samples and compiled the resource materials. OSMS and CHMN performed the test and data analysis under the supervision of SM and JP. All authors read and approved the final manuscript.
